# Association between inflammatory airway disease of horses and exposure to respiratory viruses: a case control study

**DOI:** 10.1186/s40248-015-0030-3

**Published:** 2015-11-03

**Authors:** Ashley Houtsma, Daniela Bedenice, Nicola Pusterla, Brenna Pugliese, Samantha Mapes, Andrew M Hoffman, Julia Paxson, Elizabeth Rozanski, Jean Mukherjee, Margaret Wigley, Melissa R. Mazan

**Affiliations:** Cummings School of Veterinary Medicine, Tufts University, North Grafton, MA USA; University of California, Davis, Davis, CA USA; The College of the Holy Cross, Worcester, USA

**Keywords:** Asthma, Bronchoalveolar lavage, Equine rhinitis virus, Equine herpesvirus-2, Pulmonary function testing

## Abstract

**Background:**

Inflammatory airway disease (IAD) in horses, similar to asthma in humans, is a common cause of chronic poor respiratory health and exercise intolerance due to airway inflammation and exaggerated airway constrictive responses. Human rhinovirus is an important trigger for the development of asthma; a similar role for viral respiratory disease in equine IAD has not been established yet.

**Methods:**

In a case–control study, horses with IAD (*n* = 24) were compared to control animals from comparable stabling environments (*n* = 14). Horses were classified using pulmonary function testing and bronchoalveolar lavage. PCR for equine rhinitis virus A and B (ERAV, ERBV), influenza virus (EIV), and herpesviruses 2, 4, and 5 (EHV-2, EHV-4, EHV-5) was performed on nasal swab, buffy coat from whole blood, and cells from BAL fluid (BALF), and serology were performed. Categorical variables were compared between IAD and control using Fisher’s exact test; continuous variables were compared with an independent t-test. For all analyses, a value of *P* <0.05 was considered significant.

**Results:**

There was a significant association between diagnosis of IAD and history of cough (*P* = 0.001) and exercise intolerance (*P* = 0.003) but not between nasal discharge and IAD. Horses with IAD were significantly more likely to have a positive titer to ERAV (68 %) vs. control horses (32 %). Horses with IAD had higher log-transformed titers to ERAV than did controls (2.28 ± 0.18 v.1.50 ± 0.25, *P* = 0.038). There was a significant association between nasal shedding (positive PCR) of EHV-2 and diagnosis of IAD (*P* = 0.002).

**Conclusions:**

IAD remains a persistent problem in the equine population and has strong similarities to the human disease, asthma, for which viral infection is an important trigger. The association between viral respiratory infection and development or exacerbation of IAD in this study suggests that viral infection may contribute to IAD susceptibility; there is, therefore, merit in further investigation into the relationship between respiratory virus exposure and development of IAD.

## Background

Inflammatory airway disease (IAD) has been identified as a common cause of respiratory abnormalities and poor performance in horses. IAD is characterized by airway inflammation and airway hyperresponsiveness [1] as well as exercise intolerance, variable coughing, nasal discharge, and increased mucus in the airways [2], and affects a large percentage of stabled horses, resulting in chronic poor respiratory health and poor performance [3–5]. The pathophysiology of IAD has not been fully elucidated and is thought to be influenced by both environmental and genetic factors [6]. Although exposure to environmental particulates and endotoxin likely plays a large role in the induction of IAD [7], a role for viral infection in lower airway inflammation has been proposed [5]. Humans suffer from a similar disease, asthma, and respiratory viruses have been firmly connected to the induction and exacerbation of asthma [8]. Human rhinovirus (HRV), genus *enteroviridae* of the family *picornaviridae*, is the predominant cause of the common cold and is the most common viral cause of exacerbation of wheezing in patients with asthma [9]. Equine rhinitis virus (ERV, until recently classified as a rhinovirus), is also a picornavirus with the A variant (ERAV) in the *Apthovirus* genus and the B variants (ERBV-1,2,3) in the *Erbovirus* genus [10], and is similarly a common cause of respiratory infection in horses [11]. The incidence of ERV in certain equine populations is high, with 43 % of Australian racehorses seroconverting to ERAV within 7 months of entering a training barn [10], however, a role for equine rhinitis viruses in poor performance has yet to be proved [12].

Herpesviruses have also been implicated in poor performance in horses: past studies have associated equine herpesvirus-1 (EHV-1) and equine herpesvirus-4 (EHV-4) infection with IAD, but they have only employed serology [13]. More recently, naturally occurring equine herpesvirus-2 (EHV-2) infection confirmed by PCR has been associated with increased numbers of neutrophils in the respiratory secretions [14] and inoculation with EHV-2 has been shown to result in prolonged (3-week) airway inflammation [15]. Our current study evaluates horses which fulfill a case definition of recent onset or exacerbation of IAD (within the past month) versus control horses for evidence of exposure or active infection with common respiratory viruses including ERAV, ERBV, EHV-2, EHV-4, equine herpesvirus-5 (EHV-5), and equine influenza virus (EIV) measured by PCR of bronchoalveolar lavage fluid cell pellets, peripheral blood buffy coat, and nasal swab, and by serologic detection of viral antibodies. We hypothesized that recent infection with equine rhinitis viruses or other respiratory viruses, similar to respiratory viruses and asthma, is associated with exacerbation or induction of equine IAD.

## Methods

In accordance with the Consensus on IAD by the American College of Veterinary Internal Medicine [6], criteria for horses with IAD included a history compatible with non-infectious inflammatory airway disease, including cough, exercise intolerance/poor performance, or nasal discharge, as well as recent (within 4 weeks) onset or exacerbation of signs. Further inclusion criteria upon diagnostic sampling included inflammatory BALF cytology (PMNs > 5 % OR mast cells > 2 % OR both). Exclusion criteria for IAD horses included a history more suggestive of recurrent airway obstruction (RAO), including obvious respiratory effort at rest and repeatable episodes of respiratory difficulty when exposed to dusty or moldy environments, recent fever (within 4 weeks), or evidence of bacterial infection on BALF cytology. Control horses were included only if they did not present any history or evidence on physical examination of respiratory disease including cough, nasal discharge, or respiratory effort, or fever for any reason within the past 4 weeks. Control horses were also required to have normal BALF cytology and no evidence of airway hyperresponsiveness or airway obstruction. Horses for this study included those presented to the Hospital for Large Animals at the Cummings School of Veterinary Medicine at Tufts University as well as those seen in the field. In order to standardize environmental conditions, horses were only included in the study if they were stabled at night and turned out during the day, and were fed a combination of hay and concentrate. Horses came from barns with a minimum of 2 horses and a maximum of 30 horses. One barn provided 4 horses, 2 of which had IAD and 2 of which were controls. One barn provided 3 controls, and one barn provided 2 controls. All other horses, both IAD and control, were from separate barns. Both IAD and control horses were sampled throughout the year at similar frequencies, although more IAD than control horses were sampled at all times of year. All horses were pleasure horses or lower-level sport horses. We sampled 46 horses, including 26 horses with a history compatible with IAD and 18 horses without an owner or referring veterinarian complaint of suspected respiratory disease. Of the horses with suspected IAD, 2 had a history or signs on physical examination or lung function testing that were compatible with RAO; these horses were excluded but the other 24 were included in this study. Out of the 18 potential control horses, 3 were lost due to positive histamine bronchoprovocation tests, and one due to presence of guttural pouch infection.

### Testing overview

Horses first underwent physical examination including use of a rebreathing bag to enhance auscultation; subsequently, baseline lung function testing and histamine bronchoprovocation (HBP) testing or albuterol challenge were performed followed by bronchoalveolar lavage. Pulmonary function testing required from 20 to 45 min depending on the method used and airway responsiveness of the horse (e.g., forced oscillatory mechanics (FOM) is performed more quickly than flowmetric plethysmography (FP, Open Pleth), and histamine bronchoprovocation is truncated in horses with more reactive airways regardless of method used.) Horses with total respiratory system resistance (R_RS_) > 1.5 cmH_2_O/l/s were given 5 puffs of albuterol^1^ via Aerohippus^2^ [16] and lung function was re-measured after 20 min. A positive response was considered a 25 % or greater decrease in R_RS_. After lung function testing, bronchoalveolar lavage, nasal swab, and blood draw were performed as described below, taking in total approximately 30 min. The entire procedure took from 1–1.5 h for each horse.

### Bronchoalveolar lavage and slide preparation

BAL was performed with either a commercial cuffed BAL tube^3^ or by bronchoscopy, and 2 aliquots of 250 ml warmed saline, as described previously [1]. The 2 samples were pooled, and slides were prepared by cytocentrifugation or by centrifugation followed by making a thin smear with the sediment. In addition, the BAL fluid was kept on ice and processed within 4 hours for PCR identification of selected viruses. BAL slides were stained with modified Wright stain and Toluidine Blue^4^, the latter for enumeration of mast cells [17]. Cells were classified by one of the authors (MRM) as percentage of macrophages, lymphocytes, neutrophils (PMN), eosinophils, and mast cells by classifying a minimum of 500 cells (1,000× magnification).

### Pulmonary function testing

Each horse underwent baseline pulmonary function testing followed by either histamine bronchoprovocation or albuterol challenge using either flowmetric plethysmography or forced oscillatory mechanics.

Flowmetric plethysmography was performed with a commercial system^5^ as described previously [16]. Briefly, each horse was sedated (detomidine^6^ 0.01-0.02 mg/kg BW IV), and fitted with an airtight mask, pneumotachograph^7^, and 2 respiratory inductance bands placed at the 11th intercostal space and just behind the last rib. The system was calibrated according to the manufacturer’s instructions. Measurement of airway obstruction was calculated by the software by subtracting the flow signal generated by the thoracic and abdominal volume change from the air flow measured by the pneumotachograph at peak expiration, termed the delta flow (DF). Delta flow increases with bronchoconstriction, as the expected airflow through the pneumotachograph is less than the observed volume shift over time as measured by the abdominal and thoracic bands.

Monosinusoidal forced oscillatory mechanics (FOM, 1-3Hz) was performed as previously described [18]. In brief, total respiratory system resistance (R_RS_) was measured in sedated horses (0.4–0.6 mg/kg BW xylazine^8^ IV). Sinusoidal flow (1–3 Hz) was generated using compressed air (75 psi) released through a proportional pneumatic valve^9^ and superimposed over the horse’s spontaneous breathing frequency via a latex sealed low dead space facemask. Flow at the mask opening was measured with a pneumotachograph and the difference between mask and atmospheric pressures was recorded with a differential pressure transducer^10^. Total respiratory impedance and resultant respiratory resistance were calculated as described previously [18].

### Histamine bronchoprovocation

Airway hyperresponsiveness was assessed via histamine bronchoprovocation as previously described [2]. In short, after baseline measurements, either total R_RS_ or DF were measured after nebulization with 0.9 % saline^11^ (as negative control), and incremental concentrations of histamine^12^ (2,4,8,16 ,and 32 mg/ml). Sensitivity to histamine was determined as the dose (mg/ml) required to elicit a 75 % increase in R_RS_ using FOM or a 50 % increase in DF using flowmetric plethysmography by interpolation of the dose–response curve [19, 20]. For either method, testing was halted if clinical reaction (increased respiratory rate or effort, repeated coughing) was detected in the horse and the histamine dose at which the clinical reaction occurred was considered to be the reactive dose.

### Albuterol challenge

In horses with baseline R_RS_ > 1.5 cmH_2_O/l/s (3 animals), albuterol was given via metered dose inhaler using the Aerohippus (5 puffs, 90 ucg/puff) to elicit bronchodilation. A positive response was considered ≥ 25 % decrease in R_RS_. No horse tested via flowmetric plethysmography had a DF greater than 3.5 l/s, therefore all underwent HBP [20].

### Sample preparation

#### Serum

Collection tubes of whole blood were allowed to clot for 30 min after sampling, and were centrifuged at 3,000 × g for 10 min at 4 C. The serum was separated and stored at −80 °C until submission for serologic testing.

#### PCR

All samples were kept on ice until they were processed. Four collection tubes of BALF were centrifuged at 500xg for 10 min at 4 °C. Cell pellets were isolated and stored in RNAprotect^13^, and the nasal swab was placed in viral culture medium^14^. Blood in EDTA was centrifuged at 3,000 × g for 10 min at 4 °C, and the buffy coat was removed and stored in RNAprotect. All samples were held at −80 °C until submission for PCR.

Nucleic acid extraction from whole blood, nasal secretions and bronchoalveolar lavage fluid was performed using an automated nucleic acid extraction system^15^ according to the manufacturer’s recommendations.

Total RNA was purified as follows: 20 ul of each freshly extracted nucleic acid sample containing genomic DNA (gDNA) and total RNA was digested with DNAse for 60 min at follows: 20 ul of each freshly extracted nucleic acid sample (containing genomic DNA (gDNA) and total RNA) was digested with DNAse for 60 min at 37 °C to remove gDNA. DNase was inactivated at 95 °C for 5 min. Complementary DNA (cDNA) from each sample was synthesized using 50 U SuperScript III^16^ in a 40 ul final volume containing 50 mM Tris–HCl, pH 8.3, 50 mM KCl, 8 mM MgCl2, 0.5 mM dNTPs, 40 U RNAsin, 0.5 mM dithiothreitol (DTT) and 600 ng random hexadeoxyribonucleotide (pd(N)6) primers (random hexamers^17^). The reaction was performed at 50 °C for 60 min. After inactivation at 95 °C for 5 min, the reaction volume was adjusted to 100 ul with nuclease-free water. Whole blood, nasal secretions and bronchoalveolar lavage fluid was assayed for the presence of EIV, EHV-2, EHV-4, EHV-5, ERAV and ERBV using previously reported qPCR assays [21, 22]. To determine the sample quality and efficiency of nucleic acid extraction we analyzed all samples for the presence of the housekeeping gene equine glyceraldehyde-3-phosphate dehydrogenase (eGAPDH), as previously described [23].

### Serologic testing

Evidence of viral infection was assessed through serological examination of single blood samples by using serum neutralization tests for EHV-2, EHV-4, ERAV-1, and ERBV-2, and hemagglutination inhibition tests for EIV-A. Because of inability to determine vaccinal vs infectious cause of positive titers for EHV-4 and EIV-A when only one time point was considered, we only analyzed serology for EHV-2, ERAV-1, and ERBV-2, none of which had available vaccines at the time of the study. Serologic testing was not performed for EHV-5. All serologic testing was performed at Cornell Animal Health Diagnostics Center. A positive titer was determined according to guidelines from the Cornell Animal Health Diagnostics Center (personal communication, Dr. Edward Dubovi), as follows: Titers considered consistent with infection or exposure were defined as follows: ≥8 for EHV-2, ≥96 for ERAV, and ≥32 for ERBV.

### Statistical analysis

All continuous variables were examined graphically for normality. Non-normally distributed continuous variables are described with median (range), and normally distributed continuous variables are described with mean ± SEM. Continuous variables that were not normally distributed were transformed mathematically prior to analysis, and described with mean ± SEM. Categorical variables were compared between horses with and without IAD using Fisher’s exact test. Continuous variables were compared between horses with and without IAD using independent t-test. For all analyses, a value of *P* < 0.05 was considered significant. Data analyses were performed using commercial statistical software^18^.

## Results

A total of 24 horses with a diagnosis of IAD based on the previously mentioned criteria, and 14 asymptomatic control horses were included in the study. The mean age of IAD-affected horses was 16.2 years ± 0.9, and the mean age of the controls was 14.5 years, ± 1.9. Breeds accounting for 15–23 % of horses were Quarterhorse and Warmblood, breeds accounting for 10–13 % of horses were Grade and Morgan, and Standardbred, Draft, Thoroughbred, Appaloosa, and Paso Fino each accounted for 5 % or less. There were no differences between the IAD and CTL populations for age, sex, or breed (Table 1). Horses with evidence of airway inflammation or abnormal lung function were excluded from the control population; accordingly, IAD horses had significantly greater numbers of neutrophils and mast cells in the BALF, and PC_75_R_RS_/PC_50_DF were significantly lower (Table 1). Only 5/24 IAD horses had elevated percentages of mast cells with normal percentages of neutrophils, 6/24 had elevated percentages of both mast cells and neutrophils, and the remainder, 13/24, had only elevated percentages of neutrophils. The majority of IAD horses had both abnormal BALF cytology and abnormal pulmonary function tests (21/24). The 3/24 IAD horses with normal PFTs had normal PMN percentages on BALF with elevated mast cell percentages. There was a strong association between elevated percentages of BALF neutrophils or mast cells and abnormal lung function (either airway hyperresponsiveness (AWHR) or response to albuterol challenge), *P* < 0.001. The majority of horses with IAD had an owner complaint of cough (75 %), or exercise intolerance/poor performance (83 %), whereas only a small number had an owner complaint of nasal discharge (17 %). There was a significant association between history of cough (*P* = 0.001) and exercise intolerance (*P* = 0.003) and diagnosis of IAD, but not between nasal discharge and IAD (Table 1). There was no association between history of cough or exercise intolerance and PCR-detection of any virus.

**Table 1:** Descriptive statistics for the study population

Group	Number	Age	Clinical signs (total number, percent)			BAL		Pulmonary function tests	
			Cough	Exercise intolerance/poor performance	Nasal discharge	PMN	Mast	Histamine (PC75R_RS_ or PC50DF)	Albuterol (>25 % decrease)
Control	14	14.5 ± 1.99 (5–26)	0 (0)	4 (29 %)	0	2.7 ± 1.6 (0–5)	0.5 ± 1.6 (0.0–1.8)	13.48 ± 1.17 (6.7–24.0)	None
IAD	24	16.2 ± 0.9 (9–25)	18 (75 %)	20 (83 %)	4 (16.7 %)	22.5 ± 4.4 (0.0–85.0)	3.29 ± 0.7 (0.0–16.0)	5.25 ± 0.90 (0.5–16.9)	3

All horses had history, clinical examination, bronchoalveolar lavage, and baseline pulmonary function testing performed. The majority of horses had HBP performed; 3 horses with R_RS_ > 1.5 cmH_2_O/l/s had albuterol challenge performed with at least 25 % decrease in RRS. Cough, exercise intolerance/poor performance, and nasal discharge reflect the owner complaint. PMN and mast refer to the neutrophil and mast cell percentages found on cytology of BALF. Histamine refers to the results of a histamine bronchoprovocation test using either FOM or Open Pleth. A lower number indicates greater reactivity of the airways. Albuterol indicates that the horse was given albuterol after baseline pulmonary function testing with at least 25 % decrease in respiratory resistance

**Fig. 1:**
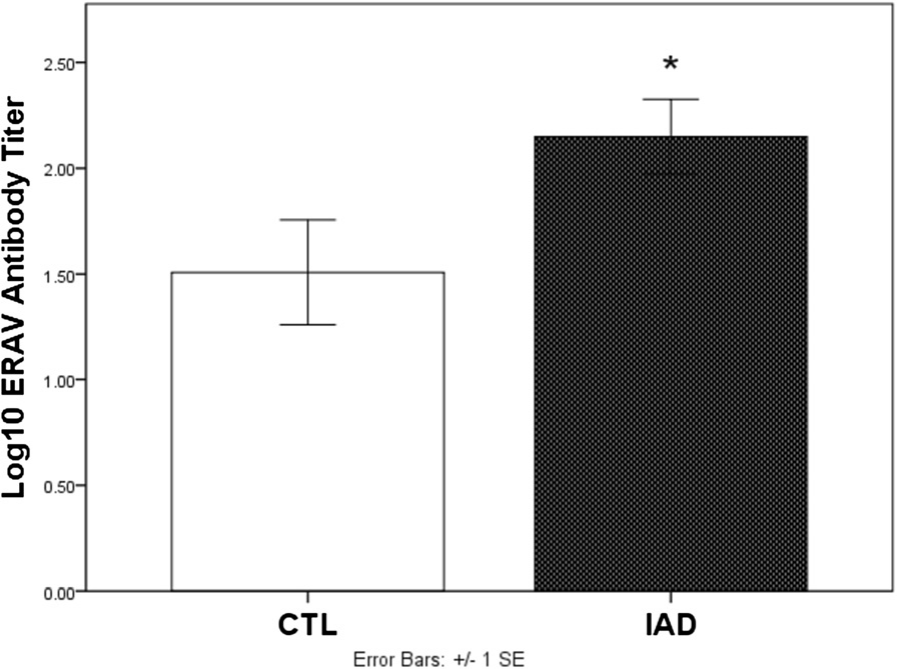
Serum neutralizing antibodies to ERAV were measured in 22/24 horses with a diagnosis of IAD and in 13/14 control horses. The antibody titers were log-transformed and expressed as mean ± standard error of the mean. Student’s paired t-test was used and found that ERAV titers in the IAD group were significantly higher than in the CTL group (2.28 ± 0.18 v. 1.50 ± 0.25). *Indicates a significant difference between groups (*P* = 0.038)

Serology was available for 22/24 horses with IAD and 13/14 control horses. There was a high seroprevalence for ERAV (54 %), ERBV (89 %), and EHV-2 (40 %) in the entire population, but horses with IAD more frequently had positive titers to ERAV (68 %) v. control horses (31 %) (*P* < 0.03) (Table 2). Horses with a diagnosis of IAD had higher log-transformed titers to ERAV than did control horses. (2.28 ± 0.18 v. 1.50 ± 0.25, *P* = 0.038) (Fig. 1).

**Table 2:** Seroprevalence for ERAV, ERBV, and EHV2 in the study population

Exposure	ERAV		ERBV		EHV2	
	IAD	CTL	IAD	CTL	IAD	CTL
Positive	15	4	20	11	8	6
Negative	7	9	2	2	14	7
% Positive	*68	31	91	85	36	46
Seroprevalence	54		89		40	

Serology was performed on 22/24 horses in the IAD group, serum samples were unavailable for 2 horses. Serology was performed in 13/14 horses in the CTL group, serum samples were unavailable for 1 horse. Percent positive for IAD and CTL groups are given for each virus ; seroprevalence for the population as a whole is also given. Titers considered consistent with infection or exposure were defined as follows: ≥8 for EHV-2, ≥96 for ERAV, and ≥32 for ERBV *Horses with IAD were significantly more likely to have a positive titer (>96) to ERAV (*P* < 0.03)

PCR was available for all horses in the study. No sample for any horse was positive for EHV-4 or ERAV. Out of 38 horses tested, only 12 were PCR positive on any sample for any virus, 9/12 of these horses were in the IAD group. Six IAD horses were positive for EHV-2 on nasal swab, but no control horse was positive (Table 3). There was a significant association between nasal shedding (positive PCR) of EHV-2 and diagnosis of IAD (*P* = 0.002) (Table 3). There were no associations between airway neutrophilia or mastocytosis and positive PCR status or between AWHR and positive PCR status.

**Table 3:** Respiratory virus and sample site for horses positive on PCR testing

Horse	Disease status	EHV-2	EHV-5	ERBV	EIV
1	CTL				NS, BAL
2	CTL	BC	BAL		
3	CTL			BAL	
4	IAD	NS			NS
5	IAD	NS			
6	IAD	BC		NS	
7	IAD	NS			NS, BAL, BC
8	IAD	BC, NS			
9	IAD				BAL
10	IAD	NS		NS,BAL	BAL
11	IAD	NS			BC
12	IAD		BAL		

This table only shows the horses that were positive on at least one sample when PCR was used to test for presence of virus. PCR for ERAV and EHV-4 is not shown as there were no positive samples on PCR. 12 horses had at least one PCR positive sample. The table shows the individual horses who were shedding EHV-2

*BC* buffy coat, *BAL* bronchoalveolar lavage cell pellet, *NS* nasal swab

## Discussion

This study was designed to determine if current or recent infection with equine rhinitis virus or other respiratory viruses plays a role in the development or worsening of IAD. In humans, wheezing episodes in early life due to infection with HRV significantly increase the chances of a diagnosis of the similar disease, asthma, at six years of age [24], and infections later in life are associated with worsening of asthma [25]. Herpesviruses are less firmly linked to wheezing episodes in early life in human infants [25], but may be associated with development of other atopic disease through immune dysregulation [26]. Despite the strong association of HRV with asthma, recent data suggest that development of asthma later in life may be dependent on the number of viral respiratory infection episodes rather than the type of virus [27]. In all, the evidence firmly inculpates respiratory virus as an important determinant of development of asthma. In contrast, although researchers and clinicians have long suspected that respiratory viruses are important to the development or exacerbation of IAD in horses [5, 28, 29], there is a paucity of data in the veterinary literature definitively making this link, and this lack was identified in the last ACVIM Consensus Statement on IAD [6].

Our study showed that there was a significant association between diagnosis of IAD and seropositivity to ERAV, with 68 % of horses with IAD versus 31 % of control horses having a titer ≥96 (Table 2). A vaccine for ERAV has only recently been available commercially, and was not available during the study time period; thus, these titers reflect the natural infection status of the horses. Not only did more IAD than CTL horses have positive titers to ERAV, but IAD horses also had significantly higher log-transformed titers for ERAV than did control horses (Fig. 1). Although serology for EHV-2 failed to distinguish IAD from CTL horses (Table 2), and few horses were PCR positive on any sample to any virus (Table 3), nonetheless there was a significant association between EHV-2 shedding (positive nasal swab on PCR) and diagnosis of IAD (Table 3), primarily because the only horses positive on nasal swab for EHV2 had a diagnosis of IAD. Although these associations do not provide any causal relationships, they do provide us some justification for further investigation and discussion.

In considering the serologic evidence for the role of ERAV in the etiology of IAD, it is important to consider what has previously been termed a positive titer and used to report seroprevalences. Our study employed the cut-point of 96 for ERAV using the guidelines of the laboratory in which the serological testing was performed (Edward Dubovi, personal communication), and was concordant with that used in a study in which seropositive horses had titers of ≥100 [30]. In contrast, in other studies, a positive serum-neutralizing titer has been considered >2 [31], and >10 [10] although it was noted that in older horses titers were in the range of >512 . In a recent experimental study, all ponies prior to infection had serum neutralizing titers for ERAV <2, rising to 64 on day 7 after infection [32] whereas in a suspected natural outbreak, titers of 1,024 were seen [33]. Although there is variability in the designation of a positive titer, nonetheless, our cut-point is within the range of those previously reported. In addition, both age and geography seem to be important in determining seroprevalence for ERAV, with titers lower in younger horses and higher in older horses; as our horses were not young, we would expect their titers to be in the higher range [31]. In contrast, the majority of studies finds that seroprevalence to ERBV is high in multiple age groups [34], similar to our findings (Table 2).

There are varying reports of active disease based on virus isolation and PCR for ERVs depending on the methodology used, whereas there is a consensus that HRV is ubiquitous in human populations [35]. Although PCR is more sensitive than virus isolation, in a recent study of over 200 cases of suspected naturally occurring viral respiratory disease only 11 % of those that were negative for ERAV on virus isolation were positive on PCR [36]. Our study, in which no horse was PCR positive for ERAV and only 3/38 horses had nasal secretions positive for ERBV (Table 3), was similar to one recently reported in which no horses with acute respiratory disease had PCR-positive nasal swab, and ERBV was found in the nasal secretions of only 2.7 % of horses [21]. A year-long longitudinal study in young Standardbreds likewise found a small number of horses PCR positive for any respiratory virus on nasal swab [12]. Positive PCR identification of the other respiratory viruses (whether on BALF cell pellet, nasal swab, or buffy coat) was found with relatively low frequency in our study [Table 3]. This low prevalence of PCR positive results likely reflects the variable natural course of disease. Similarly to HRV in humans, ERAV is cleared quickly from the equine respiratory system (within 2 weeks) [11]. Despite this rapid clearance, high antibody titers are still present at 21 to 35 days after infection with ERAV [11, 33]. This is most likely the reason that, in contrast to our expectation that PCR detection of virus would be more effective in providing the link between respiratory viral disease and diagnosis of IAD, instead, due to sample timing, the indirect serologic evidence was more revealing.

In addition to a possible role for rhinitis viruses, our study showed that although only a small subset of horses was positive on nasal swab for EHV-2, this nonetheless was positively associated with a clinical diagnosis of IAD. EHV-2 is a slow-growing cytomegalovirus that has been reported to infect foals early in life and to have a high seroprevalence [37]. Although the pathogenicity of EHV-2 has previously been debated given that it can be recovered from both clinically affected and healthy animals [13], studies have linked its pathogenic potential to a modulation of the host immune response [38]. A recent study demonstrates that field strains of EHV-2 were detected in 50 % of horses tested, and after reactivation of latent infection using systemic corticosteroids, EHV-2 is detectable in the trachea up to 14 days [15]. Although this study and others have failed to show associations between clinical signs or tracheal neutrophils and EHV-2 [39], or indeed between EHV-2 viral load and poor performance [40] a recent study showed that inoculation with equine herpesvirus-2 results in prolonged neutrophilia in BALF despite resolution of other clinical signs, suggesting that the gammaherpesviruses may indeed play a role in the development of airway inflammation in horses [14]. Unlike HRV, there is far less evidence in human medicine for the involvement of herpesviruses in childhood wheeze or indeed development of asthma. Although serologic evidence of cytomegalovirus infection (a betaherpesvirus) was more prevalent in infants with asthma-like bronchial symptoms than in age-matched infants with no wheezing, arguing for cytomegalovirus infection playing some role in these cases [41], in a different study, having more than one herpesvirus infection before the age of three was actually *inversely* associated with asthma at age seven [42]. In horses, it has been proposed that EHV-2 modifies Il-10 [43], and may thus affect long-term respiratory responses through modulation of the immune response. On the other hand, as EHV2 has been shown to establish latency [44], it may be that the presence of active shedding is secondary to airway inflammation rather than a cause of airway inflammation. It remains to be determined if EHV-2 is one of the many possible insults that, combined, drive the equine respiratory phenotype toward IAD.

There was a relatively small number of horses testing PCR positive for any other viruses, which is likely due, as with ERAV, to the natural course of disease in comparison to the single sampling timeframe of our study. Equine influenza virus has been shown to be shed in nasal secretions of immunized horses for an average of 6–8 days after infection [45], while equine herpesvirus-1\-4 can be shed in nasal secretions for 14–75 days after infection [46, 47] and reactivated latent herpes virus infections are common. [48] A recent study showed that when subclinical viral respiratory disease was detected on nasal swab, horses had not seroconverted yet. [12]. Therefore, it is not surprising that sampling apparently healthy, non-febrile horses at a single time point yielded low numbers of positive PCR identification of respiratory virus. In fact, when the prevalence of respiratory disease in horses in New Zealand was surveyed, although EHV-2 and EHV-4 were among the most common viruses detected upon PCR, these viruses were only detected in horses with evidence of febrile respiratory illness [49]. Thus, selection bias likely also contributed to low EHV detection rates because horses that were PCR positive for a respiratory virus would more likely have a recent history of fever and malaise, and would have been excluded from the study. Nonetheless, it is of interest to clinicians that in a population of horses without history or clinical signs of current viral infection, 8/38 horses were shedding virus, and only one of those horses was in the control group. Further, nasal discharge, which is commonly seen in horses with viral respiratory infections, was not positively associated with PCR-positive virus status *or* a diagnosis of IAD (Table 1) and only one of the horses that shed virus had nasal discharge. This is in accordance with prior conclusions that subclinical infection with respiratory viruses is common among equine populations [31, 50].

The strong connections that have been established between respiratory viral infection and asthma in humans suggest that this may be a good model for understanding the relationship between similar equine respiratory viruses, airway inflammation and functional pulmonary derangements in horses. Recent studies have shown that there are multiple factors at play in the development of disease: asthmatics presenting to the emergency room, for instance, do not have higher viral loads than non-asthmatics [51], but it may be that a second hit, such as an environment high in dust mites [52], as well as the influences of genetics, diet, age, and immune responses [53], is necessary to precipitate a crisis. Moreover, there appear to be, as a recent study termed it, a panoply of ‘unique cellular immune factors’ that work in concert with HRV to result in wheezing and long-term asthma in children exposed to HRV [53], including a deficiency of the interferon response due to Th2 polarization in atopic individuals and a subsequent maladaptive immune response [53]. Likewise, the development of equine IAD appears to involve multiple different factors, including environment, in addition to the proposed role of respiratory viruses [6, 7]. There is emerging, if somewhat conflicting, evidence that some horses with IAD also have a polarized Th2 response [54], making it tempting to speculate that a maladaptive immune response may likewise contribute to the development of enhanced airway inflammation and hyperresponsiveness in horses with ERAV or other viral respiratory infection.

A recent review implicates changes in airway biology which result in initiation and progression of airway remodeling, disruption of the epithelial barrier, decreased ciliary function, and production of growth factors and metalloproteinases in HRV-associated asthma perturbations [35]. Although both HRV and the similar picornaviruses, ERAV and ERBV, are commonly associated only with relatively mild upper airway symptoms and signs including pharyngitis, nasal discharge, coughing and variable fever, both are able to infect the lower airways as well as the upper airways, causing long-term airway inflammation and potentially ciliary dysfunction with loss of clearance [32, 55, 56]. It is logical, therefore, that HRV causes reduced lung function [57] and AWHR in humans [58]. Despite the relatively quick clearance of HRV from the respiratory system, AWHR to methacholine persists in children from 5–11 weeks after natural infection with HRV [55]. Although a recent study in horses failed to find an overall increase in AWHR in affected ponies, primarily due to pre-existent AWHR in principal *and* control animals, nonetheless, individual animals did have a heightened response to histamine after infection with ERAV. [32] In our study, chi-square analysis showed a strong association between elevated percentages of BALF neutrophils or mast cells and abnormal lung function (either AWHR or response to albuterol challenge), *P* < 0.001, and the majority of IAD horses (>90 %) had evidence of abnormal lung function on pulmonary function testing. Our study concords with findings by other workers, where cough was highly associated with a diagnosis of IAD [59, 60]. However, due to our study design excluding horses with abnormal lung function from the control group, we were unable to look for associations across the whole population of horses between PCR or evidence of viral infection and abnormalities on lung function. In contrast to previous studies from our laboratory [1, 2], there was no correlation between AWHR and BALF cell percentages. This may be a reflection of our population: previous studies from our laboratory have had a significant proportion of young racehorses, whereas this study involved primarily middle-aged lower-level sport horses.

Clearly, there are limitations to this study. Although our samples were quickly placed on ice and transferred within 4 hours to the laboratory for appropriate storage, it is possible that samples may have degraded in transit. In addition, we may have had a higher percentage of horses testing positive on PCR if we had swabbed the nasopharynx rather than the nasal passages alone [30]. We were unable to use serology to investigate the relationship between IAD and viruses other than EHV-2, ERAV and ERBV, as we sampled only at one time-point, and we were thus unable to differentiate vaccinal status vs natural exposure. Had we sampled at 4-week intervals to detect rising titers, we might have detected a role for the more commonly diagnosed respiratory viruses in the development of IAD. As discussed above, it has been suggested that cumulative exposures to HRV are critical to the development of the asthmatic phenotype [35]. Although a longitudinal study of young Standardbred racehorses failed to find any associations among seroconversion, single antibody titers, or PCR positivity to rhinitis viruses and poor performance [12], a longitudinal study of respiratory viral disease in older performance horses may be necessary to adequately parse out the connection to development of the IAD phenotype which may develop with time and repeated insult to the respiratory system. Because environment is thought to be one of the most important of the possible repeated insults to the equine respiratory system, we standardized environment as much as possible by ensuring that horses came from very similar environments, namely a combination of stall and turnout, and that they had similar bedding and similar feed. We also ensured that control horses came from multiple different barns, therefore rendering the possibility of infection with respiratory virus more random. Nonetheless, in an ideal experiment, exposure to respiratory virus would be the only intervention made in horses housed in identical environments, thus rendering any outcome more obvious and clear. Moreover, there is a strong heritable component to asthma in children as well as mutations that may enhance viral binding in asthmatic airways [61]; no heritable component has yet been demonstrated for IAD in horses but there is some evidence for genetics to play a role in the more severe disease, RAO [62]. Future investigations into the genetics of IAD will be necessary eventually to determine if genetics and viral respiratory infection work together to create chronic disease.

## Conclusion

In conclusion, this study found that a greater percentage of horses with diagnosis of recent onset or exacerbation of IAD defined by appropriate clinical history, BALF cytology and PFTs had positive titers to ERAV than did control horses, and horses with a diagnosis of IAD had higher log-transformed titers to ERAV than controls. In addition, a diagnosis of IAD was associated with nasal shedding of EHV-2 (positive nasal swab PCR). The authors recognize that these findings are associations without having evidence of causation; nonetheless, this study provides an intriguing possible link between viral respiratory disease and exacerbation or onset of IAD. IAD remains a persistent problem in the equine population and has strong similarities to the human disease, asthma, the development or exacerbation of which is strongly associated with viral respiratory disease. Our study suggests that there is merit in further investigation of the role of viral respiratory disease in initiation or exacerbation of IAD in order to better understand disease in both horses and humans.

## Abbreviations

AWHR: Airway hyperresponsiveness
BAL: Bronchoalveolar lavage
BALF: Bronchoalveolar lavage fluid
DF: Delta flow
FOM: Forced oscillatory mechanics
FP: Flowmetric plethysmography
HRV: Human rhino virus
HBP: Histamine bronchoprovocation
IAD: Inflammatory airway disease
PMN: Polymorphonuclear leukocyte
RAO: Recurrent airway obstruction
R_RS_: Total respiratory system resistance

## Footnotes

1 Ventolin HFA, GlaxoSmithKline, Philadelphia, PA

2 Aerohippus, Trudell Medical International, London, Ontario, Canada.

3 Bivona Medical Technologies, Gary, IL

4 Toluidine Blue, Polyscientific, Bayshore, NY

5 Open Pleth, Ambulatory Monitoring Inc, Ardsley, NY

6 Dormosedan, Orion Pharma, Espoo, Finland

7 Fleisch, No 4, OEM Medical, Lenoir, NC

8 AnaSed, Akorn Inc, Decatur, IL

9 Proportional valve No. 602 00001, Joucomatic, Rueil, France

10 DP45-28, Validyne Engineering, Northridge, CA

11 0.9 % preservative-free saline, Hospira Inc, Lake Forest, IL

12 Histamine diphosphate monohydrate, MP Biomed, Solon, OH

13 RNA protect, QIAGEN

14 Viral culture medium

15 CAS-1820 X-tractor Gene, Corbett Life Science

16 SuperScript III reverse transcriptase, Invitrogen, Grand Island, NY

17 Random hexamers primers, Invitrogen, Grand Island, NY

18 SPSS v. 13.0, SPSS Corp, Chicago, IL
